# Supplementary material for: Mycobacterium tuberculosis Rv2005c Induces Dendritic Cell Maturation and Th1 Responses and Exhibits Immunotherapeutic Activity by Fusion with the Rv2882c Protein
Source: Vaccines (Basel). 2020 Jul 10;8(3):370. doi: 10.3390/vaccines8030370 (PMC7564171; doi:10.3390/vaccines8030370)
Supplement: Supplementary file 1 [file vaccines-08-00370-s001.pdf]

Mycobacterium tuberculosis Rv2005c induces dendritic cell maturation and Th1 responses and exhibits immunotherapeutic activity by fusion with Rv2882c protein

Back Young Woo†, Ki Won Shin†, Seunga Choi, Hye Soo Park, Kang In Lee, Han-Gyu Choi*, Hwa-Jung Kim*

[1]Department of Microbiology, of Medical Science, College of Medicine, Chungnam National University, Daejeon, Republic of Korea

† These authors contributed equally to the work.

*For correspondence

Hwa-Jung Kim, E-mail: hjukim@cnu.ac.kr,

Han-Gyu Choi, Email: ekdrms20000@cnu.ac.kr

Keywords: Tuberculosis; Hypoxia mimetic condition; chemotherapy; immunotherapy vaccine; Multifunctional T cell

Material and Method

Vaccine evaluations

In vivo Chemotherapy assay

To induce progressive pulmonary TB, mice (n = 9/group) were anesthetized with 1.2% 2,2,2-tribromoethanol (avertin), exposed via a small midline incision, and inoculated intratracheally (I.T.) with 1×10^6 CFUs of Mtb H37Ra in 50 µl saline [1]. To evaluate the protective efficacy of the Rv2005c fusion protein and the immune responses generated before and after the course of immunotherapy with this antigen and chemotherapy in Mtb-infected animals, mice were randomized (5 animals per group) and treated with Rv2005c, adjuvant alone or the combination of Rv2005c and adjuvant. The mice receiving immunotherapy with Rv2005c were administered 5 µg/dose/mouse protein emulsified in Dimethyldioctadecylammonium bromide (DDA; 250 µg/dose/mouse) and Monophosphoryl Lipid A (MPL; 25 µg/dose/mouse). The control groups were immunised with adjuvant alone. In the 'short duration' chemotherapy experiment, after 3 weeks of infection, the mice were given isoniazid (INH, 0.1 g/L) and rifampin (RIF, 0.1 g/L) in their drinking water ad libitum for 4 weeks [2]. These mice were sacrificed 3, 7 or 16 weeks later, isolated lung from immunised mice were used for CFU and ELISA analysis.

Pre-exposure vaccine assay

Mice (n = 4/group) were immunised three times at 2 week intervals subcutaneous (S.C) with 5 µg Rv2005c-DDA/MPL (250 µg / 25 µg) in a total volume of 0.2 mL. A single group of mice administrated S.C route one dose of BCG (1 x 10^4 CFU) Tokyo strain. Six weeks after the last immunisation, mice were infected with Mtb H37Ra (1 x 10^6 CFU/I.T/mice) [3]. After 6 weeks Mtb challenge, the mice were euthanized by CO_2 asphyxiation, and isolated lung from immunised or BCG vaccinated mice were used for CFU and ELISA analysis.

Prime-boost vaccine assay

C57BL/6 mice (n = 4/group) were injected subcutaneously with a single dose of 1 x 10^4 CFU of BCG Tokyo (prime), and were immunised 12 weeks later, through 3 S.C. injections administered 3 weeks apart (boosts). Each immunisation contained 5 μg of Rv2005c with 25 μg of MPL formulated in 250 μg of dimethyldioctadecylammonium (DDA) liposomes. The control group was immunised with MPL/DDA only. Immunised mice (MPL/DDA, BCG and BCG+Rv2005c/MPL/DDA) were infected with M.tb H37Ra (1 x 10^4 CFU/I.T/mice) [4]. Sixteen weeks after the infected, the mice were euthanized by CO_2 asphyxiation, and isolated lung from immunised mice were used for CFU and ELISA analysis.

.

Supplemental Figures

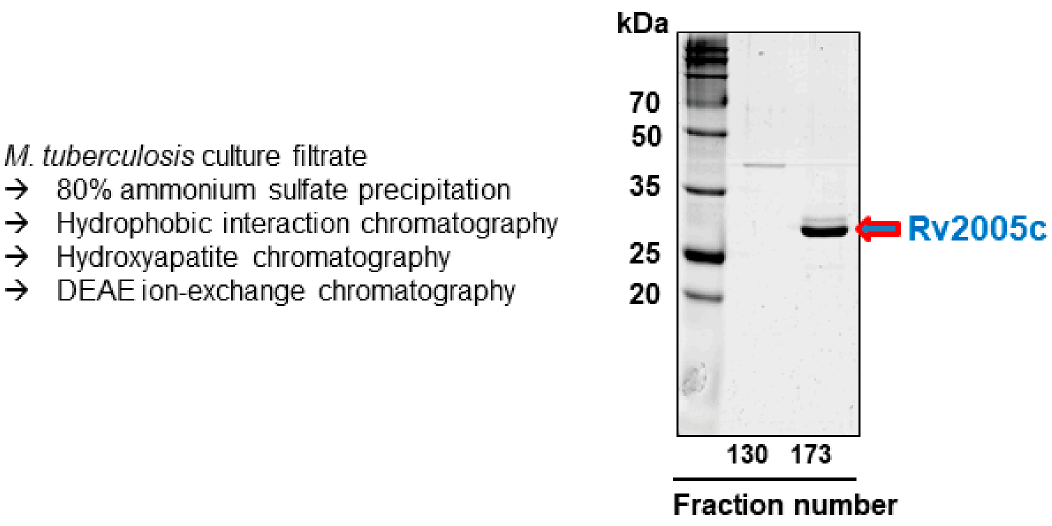

Supplemental Figure 1. Identification of Rv2005c protein among *M. tuberculosis* culture filtrate proteins (CFPs). Mtb CFPs were fractionated into 180 fractions as previously described [5]. In brief, the 80% ammonium sulfate precipitate of the CFP was initially fractionated into 8 fractions by hydrophobic interaction chromatography (HIC) and then sequentially further fractionated by hydroxyapatite chromatography (HAT) and DEAE ion-exchange chromatography (IEC). The fractions that strongly reacted with the sera from active tuberculosis patients were analyzed by SDS-PAGE with Coomassie brilliant blue staining.

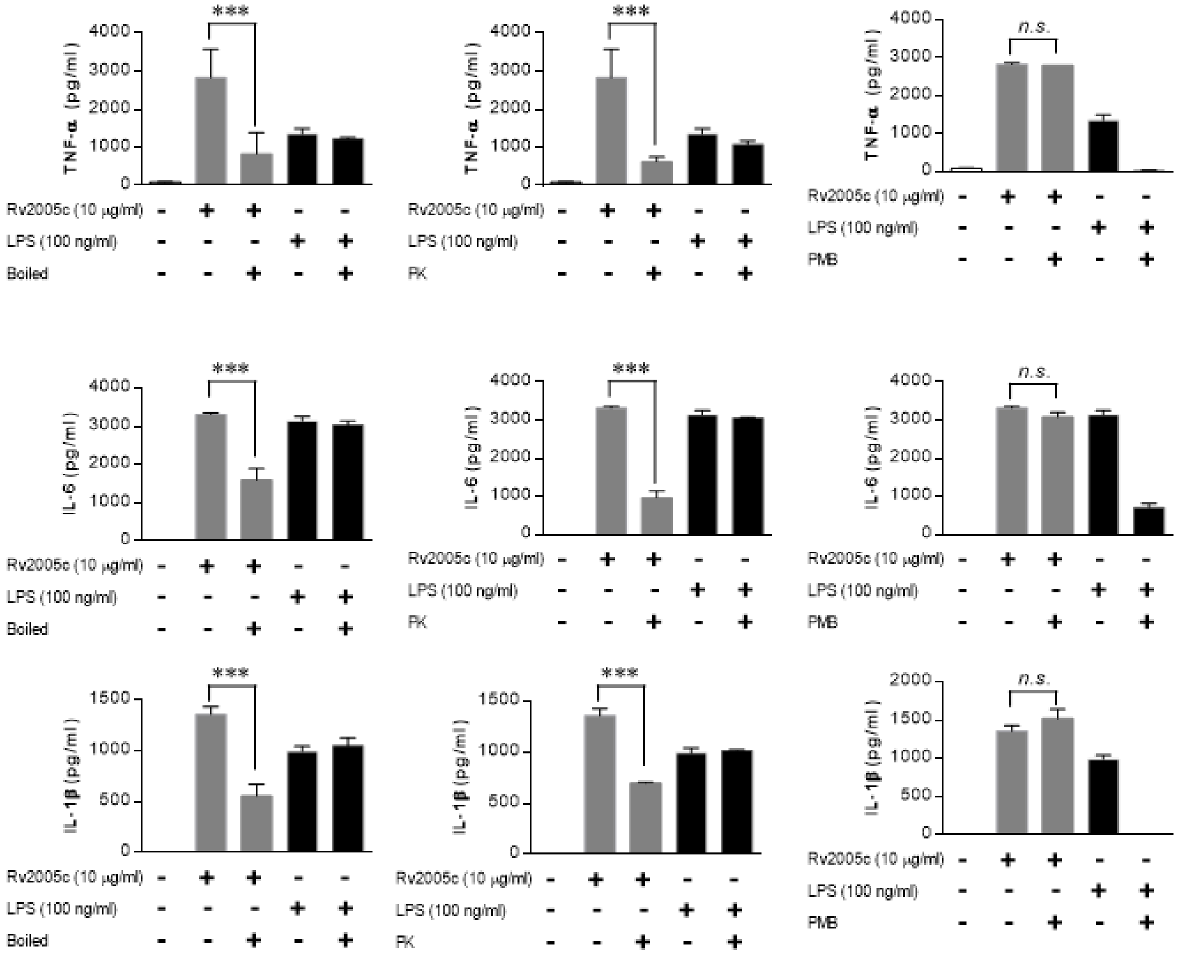

Supplemental Figure 2. Confirmation of endotoxin decontamination of purified Rv2005c. BMDCs were stimulated with Rv2005c that was denatured by boiling for 30 min at 100°C or digested with proteinase K (PK, 10 μg/mL) for 1 h at 37°C. Alternatively, DCs were pretreated with polymyxin B (50 μg/mL) for 1 h prior to stimulation of the DCs. LPS (100 ng/ml) was used as the control. After 24 h, the concentrations of TNF-α, IL-1β, and IL-6 in the culture medium were measured by ELISA. All the data are expressed as the mean ± SD (n = 3), and statistical significance (***$p < 0.001$) is indicated for the treated groups compared to the control groups, and differences that were not significant are indicated as *n.s.*

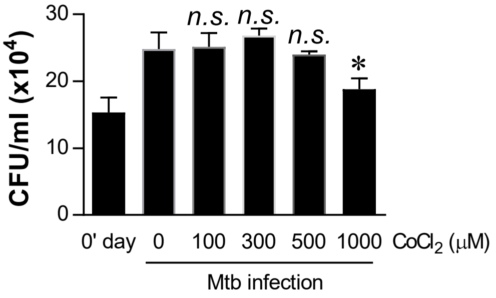

Supplemental Figure 3. Confirmation of Mtb growth inhibition by $CoCl_2$. BMDMs were infected with H37Rv at an MOI of 5 for 2 h, and then the extra bacteria were removed by washing. Then, the cells were treated with 100-1000 μM $CoCl_2$ for 8 h and lysed, and intracellular Mtb growth in the BMDMs was determined at 0 and 3 days after treatment with $CoCl_2$. All the data are expressed as the mean ± SD (n = 3). The levels of significance ($*p < 0.05$) for the BMDMs treated with $CoCl_2$ compared to those not treated with $CoCl_2$. *n.s.:* no significant difference.

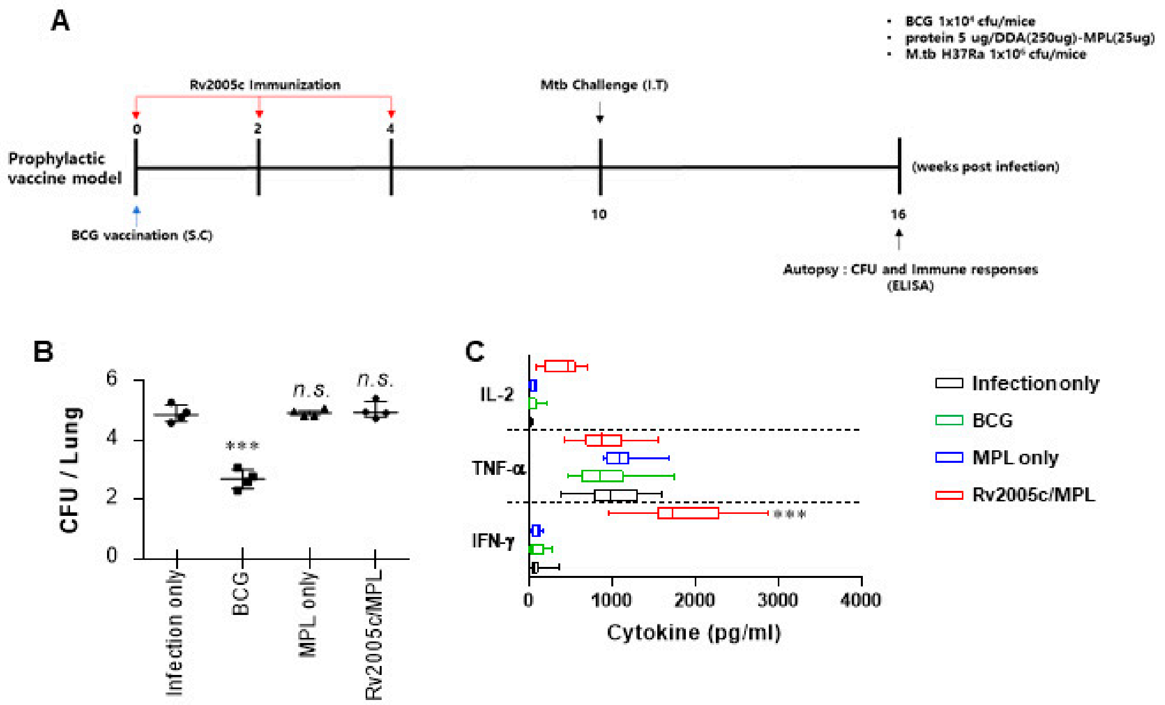

Supplemental Figure 4. Rv2005c does not induce significant prophylactic vaccine efficacy. (A) Mice received three immunisations with a two-week interval between each immunisation. Six weeks after the last immunisation, the mice were intratracheally infected with 1 x 10^6 CFU of Mtb H37Ra. Six weeks postinfection, the mice were sacrificed, and the CFU levels in the lungs were determined. (B) The bacterial loads in the lungs of mice following different treatment schedules compared to those in the control untreated mice. The results from one of two experiments that produced similar results are shown (n = 3 animals/group). ***p<0.001 compared with the infection only group. (C) The IFN-γ, TNF-α, and IL-2 production by lung cells in response to Rv2005c stimulation was measured by ELISA. ***p<0.001 compared with the infection group.

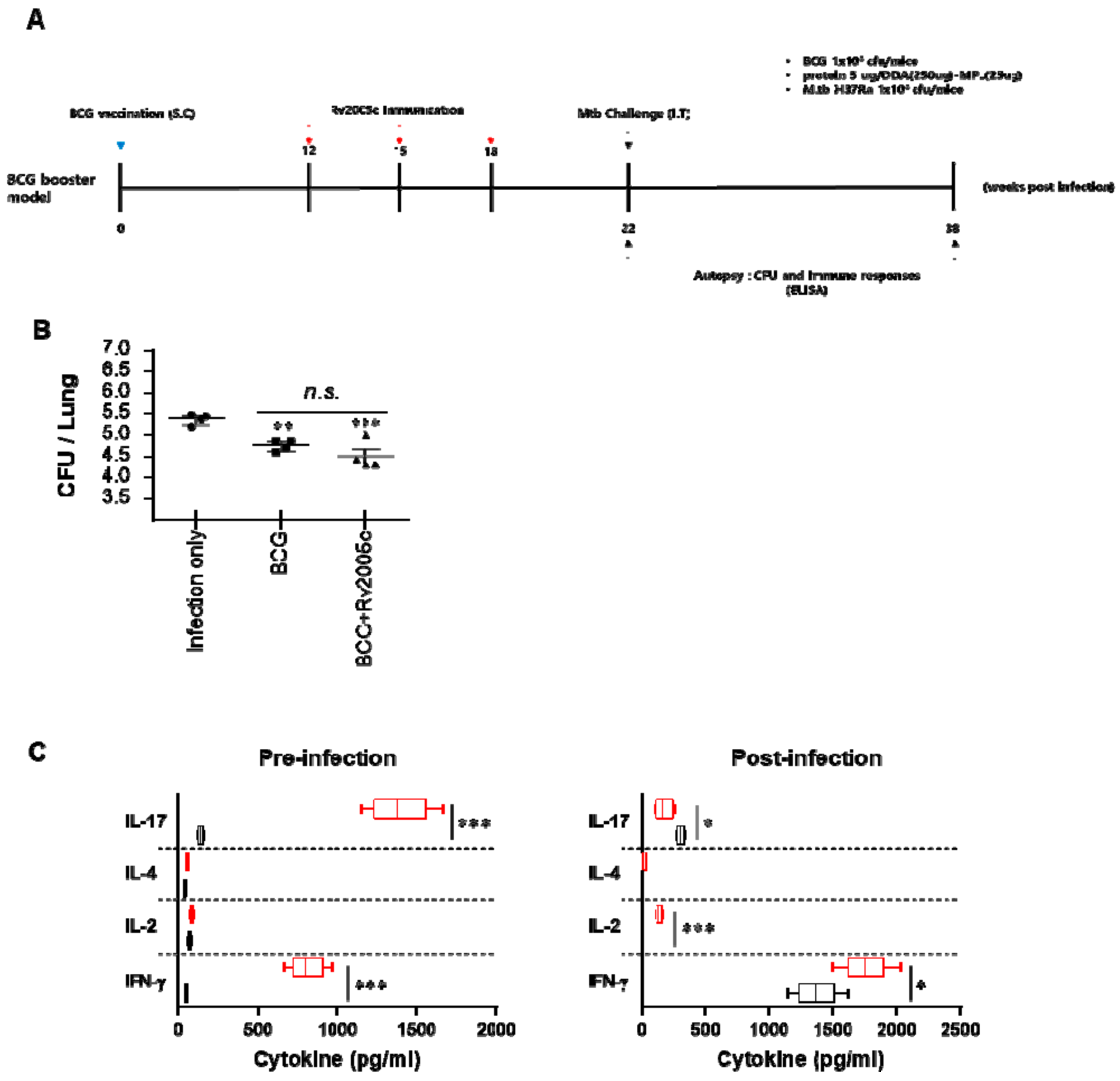

Supplemental Figure 5. Rv2005c does not induce significant BCG prime-boost vaccine efficacy. (A) Mice were first primed subcutaneously (S.C.) with 1 x 10^4 CFU BCG, and 12 weeks later, the mice were boosted with a three-week interval between each immunisation. Four weeks after the last immunisation, the mice were intratracheally infected with 1 x 10^4 CFU of Mtb H37Ra. Four weeks following the last vaccination or sixteen weeks postinfection, the mice were sacrificed, and ELISA analysis was conducted or the CFU levels in the lungs were determined. (B) The bacterial loads in the lungs of mice following different treatment schedules compared to those in the control untreated mice. The results from one of two experiments that produced similar results are shown (n = 4 animals/group). $**p<0.01$ and

$***p<0.001$ compared with the infection alone group. (C) The IFN-γ, IL-2, IL-4 and IL-2 production by lung cells in response to Rv2005c stimulation preinfection or postinfection was measured by ELISA. The data shown are the mean values ± SDs ($n = 3$); $*p < 0.05$, and $***p<0.001$ compared with the control group. Red box, untreated; black box, Rv2005c stimulated.

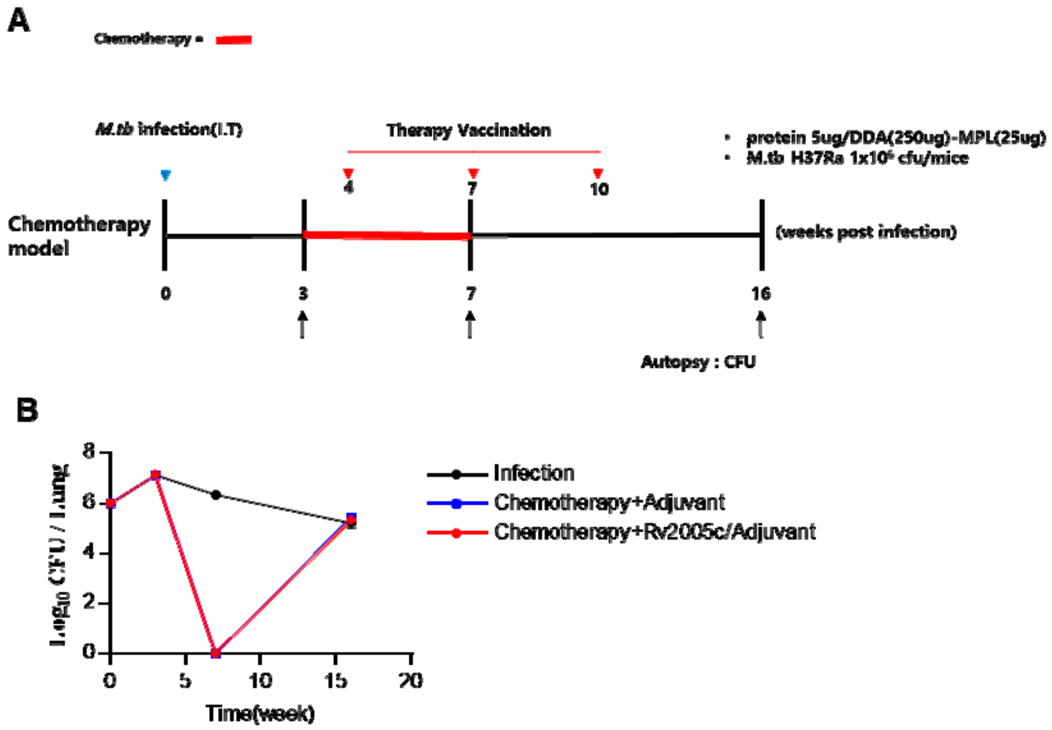

Supplemental Figure 6. The Rv2005c does not induce significant chemotherapy vaccine efficacy. (A) Schematic diagram in each panel shows the detailed treatment schedule of the drug-treated and drug + immunotherapy-treated groups. (B) The bacterial loads in the lungs of mice following different treatment schedules compared to those in the control untreated mice. Chemotherapy was given for 4 weeks. The bacterial counts are expressed as the $\log_{10}$ value of the colony forming units. The data represent the mean CFU of three mice in each group.

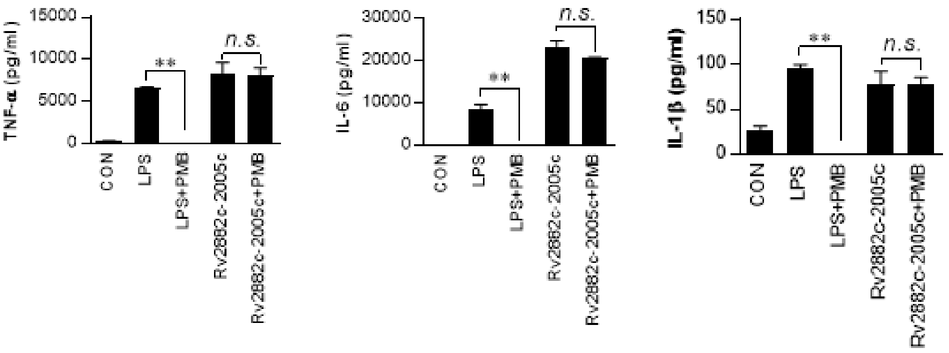

Supplemental Figure 7. Confirmation of endotoxin decontamination of purified Rv2882c-Rv2005c fused protein. DCs were pretreated with polymyxin B (50 μg/mL) for 1 h prior to stimulation with the Rv2882c-Rv2005c fused protein. LPS (100 ng/ml) was used as the control. After 24 h, the concentrations of TNF-α, IL-1β, and IL-6 in the culture medium were measured by ELISA. All the data are expressed as the mean ± SD (n = 3), and statistical significance (***$p < 0.001$) is indicated for treated groups compared to the control groups, and differences that were not significant are indicated as *n.s.*

Reference

1. Back, Y.W.; Choi, S.; Choi, H.G.; Shin, K.W.; Son, Y.J.; Paik, T.H.; Kim, H.J. Cell wall skeleton of mycobacterium bovis bcg enhances the vaccine potential of antigen 85b against tuberculosis by inducing th1 and th17 responses. *PloS one* **2019**, *14*, e0213536.
2. Botha, T.; Ryffel, B. Reactivation of latent tuberculosis by an inhibitor of inducible nitric oxide synthase in an aerosol murine model. *Immunology* **2002**, *107*, 350-357.
3. Hoang, T.; Aagaard, C.; Dietrich, J.; Cassidy, J.P.; Dolganov, G.; Schoolnik, G.K.; Lundberg, C.V.; Agger, E.M.; Andersen, P. Esat-6 (esxa) and tb10.4 (esxh) based vaccines for pre- and post-exposure tuberculosis vaccination. *PloS one* **2013**, *8*, e80579.
4. Choi, H.G.; Choi, S.; Back, Y.W.; Paik, S.; Park, H.S.; Kim, W.S.; Kim, H.; Cha, S.B.; Choi, C.H.; Shin, S.J. *et al.* Rv2299c, a novel dendritic cell-activating antigen of mycobacterium tuberculosis, fused-esat-6 subunit vaccine confers improved and durable protection against the hypervirulent strain hn878 in mice. *Oncotarget* **2017**, *8*, 19947-19967.
5. Byun, E.H.; Kim, W.S.; Shin, A.R.; Kim, J.S.; Whang, J.; Won, C.J.; Choi, Y.; Kim, S.Y.; Koh, W.J.; Kim, H.J. *et al.* Rv0315, a novel immunostimulatory antigen of mycobacterium tuberculosis, activates dendritic cells and drives th1 immune responses. *Journal of molecular medicine* **2012**, *90*, 285-298.
